# Early germline differentiation in bivalves: TDRD7 as a candidate investigational unit for *Ruditapes philippinarum* germ granule assembly

**DOI:** 10.1007/s00418-021-01983-0

**Published:** 2021-03-26

**Authors:** Beatrice Filanti, Giovanni Piccinini, Simone Bettini, Maurizio Lazzari, Valeria Franceschini, Maria Gabriella Maurizii, Liliana Milani

**Affiliations:** Department of Biological, Geological and Environmental Sciences, BiGeA, University of Bologna, Bologna, Italy

**Keywords:** Germ plasm, Tudor proteins, LOTUS domain, Primordial germ cells, Manila clam, Vasa protein

## Abstract

The germline is a key feature of sexual animals and the ways in which it separates from the soma differ widely across Metazoa. However, at least at some point during germline differentiation, some cytoplasmic supramolecular structures (collectively called germ plasm-related structures) are present and involved in its specification and/or differentiation. The factors involved in the assembly of these granular structures are various and non-ubiquitous among animals, even if some functional patterns and the presence of certain domains appear to be shared among some. For instance, the LOTUS domain is shared by Oskar, the Holometabola germ plasm master regulator, and some Tudor-family proteins assessed as being involved in the proper assembly of germ granules of different animals. Here, we looked for the presence of LOTUS-containing proteins in the transcriptome of *Ruditapes philippinarum* (Bivalvia). Such species is of particular interest because it displays annual renewal of gonads, sided by the renewal of germline differentiation pathways. Moreover, previous works have identified in its early germ cells cytoplasmic granules containing germline determinants. We selected the orthologue of TDRD7 as a candidate involved in the early steps of germline differentiation through bioinformatic predictions and immunohistological patterning (immunohistochemistry and immunofluorescence). We observed the expression of the protein in putative precursors of germline cells, upstream to the germline marker Vasa. This, added to the fact that orthologues of this protein are involved in the assembly of germ granules in mouse, zebrafish, and fly, makes it a worthy study unit for investigations on the formation of such structures in bivalves.

**Supplementary Information:**

The online version contains supplementary material available at 10.1007/s00418-021-01983-0.

## Introduction

Germ cells play a unique role in animal heredity and evolution as carriers of the genetic information across generations (Gilbert 2010). In sexual Metazoa, the hereditary information moves in two ways: within the germ cell lineage (or germline) that eventually produces gametes, providing an immortal link to the next generation; and from germ cells to somatic cells to build a new organism (Juliano et al. 2010). Somatic cells develop afresh every new generation from germ cells, but between the zygote and the future re-establishment of germline in the embryo the two lineages overlap (Niklas and Kutschera 2014). Therefore, investigating timing and mechanisms involved is a central challenge for understanding the evolutionary origin and maintenance of the crucial differentiation of the two lineages.

Solana (2013) introduced the concept of Primordial Stem Cells (PriSCs). These cells are evolutionarily conserved stem cells that act as a link of germ plasm components from the zygote to the future germline (Solana 2013). PriSCs share common features with stem cells thanks to their capacity to self-renew and differentiate into specialized cells that can have both somatic and germline potential (Xie and Spradling 2000; Li and Xie 2005; Solana 2013). At some point during development, or several times during the life of animals with gonad renewal or high regeneration potential, a PriSC gives rise to a renewed PriSC and a Primordial Germ Cell (PGC) through an asymmetric cell division. PGCs are cells in proliferative state that retain self-renewal capacities and give rise to cells with only germline potential. PGCs will then populate the gonads through mitotic proliferation and give rise to Germ Cells (GCs) and gametes by meiosis.

In the past decades, various research teams have focused on identifying and characterizing the cells that act as a link between zygote and gametes. Extensive researches have focused on the identification in model organisms of germline determinants–RNAs/proteins (e.g.: Vasa, Nanos, Piwi, Tudor; Ewen-Campen et al. 2010). Many of such determinants appear to be evolutionarily conserved through Metazoa, both for their presence in the genome and for their germline-related functions (Extavour and Akam 2003; Ewen-Campen et al. 2010; Fierro-Constaín et al. 2017). However, the timing of their expression and the level of organization at which they cluster together forming germ plasm, or germ plasm-related structures, is extremely variable in different animals (Kloc et al. 2004; Whittle and Extavour 2017). Indeed, in most model taxa, germline determinants are assembled in a differentiated region of the cytoplasm, generically called “germ plasm”, that can be observed at different stages of germline specification (Kloc et al. 2004). Cells that contain the germ plasm usually divide so that only one daughter cell inherits the differentiated cytoplasm and the germline potential with it (Extavour and Akam 2003). When such cytoplasmic differentiation is inherited directly from the oocyte/zygote to a specific cell lineage, it is usually referred to as “preformation”. This is in contrast with germline specification by “induction” that involves the presence of germline-inductive signals from neighboring cells surrounding the future germline during or after embryogenesis and it is thought to represent the ancestral mode of specification in Metazoa (Extavour and Akam 2003). However, besides the timing of their appearance and the level of involvement in germline specification, it appears that ribonucleoproteic cytoplasmic germ granules are present and fundamental for the functioning of germ cells in all animals (Voronina et al. 2011; Sengupta and Boag 2012). For this reason, germ plasm/granules characterization and study are crucial for the understanding of metazoan germline patterning.

Important factors acting in germ plasm assembly of model organisms are: Oskar (in polar granules of holometabolous insects; Ephrussi et al. 1991), Xvelo (in the Balbiani body of *Xenopus laevis*; Boke et al. 2016) and Bucky ball (orthologue of Xvelo in zebrafish; Bontems et al. 2009), with Oskar being the most studied so far. Indeed, the function of the short isoform of Oskar (there are two isoforms that differ for 139 amino acids on the N-terminal) is to promote the formation of germ plasm, being necessary and sufficient for its assembly (Jeske et al. 2015), and it acts in concert with other components, such as Vasa, Nanos and Tudor (Anne 2010). The Oskar protein is assessed to be present only in the insect lineage (Ewen-Campen et al. 2012), and it includes two functional domains: the RNA-binding domain OSKAR, and the LOTUS domain. While the former has been found so far only in Oskar proteins of insects and in Bacteria (the presence in insects is likely the result of horizontal gene transfer: Blondel et al. 2020), the latter can be found also in other germline-related proteins, such as homologues of TDRD5 and TDRD7 (Anantharaman et al. 2010; Callebaut et al. 2010). In some recent studies, it has been demonstrated that the LOTUS domain of Oskar is responsible for the dimerization of the protein and it physically interacts with Vasa to regulate Vasa DEAD-box helicase activity and to mediate its localisation in the germ plasm (Anne 2010; Jeske et al. 2017). Indeed, other indirect evidence of the LOTUS–Vasa interaction come from other LOTUS-containing proteins, such as the mouse TDRD7 and the homologues of TDRD5 and TDRD7 of *Drosophila* (Tejas and Tapas, respectively), that have been observed to co-precipitate with Vasa along with other germline components (Hosokawa et al. 2007; Patil et al. 2014). Indeed, these two LOTUS-TUDOR-containing proteins have been associated to roles in the proper assembly of cytoplasmic structures/granules in different species and in different tissues: from somatic ribonucleoproteic granules involved in mammal ocular lens formation (TDRD7; Lachke et al. 2011), to mammal male germline chromatoid bodies (TDRD7 and TDRD5; Tanaka et al. 2011; Yabuta et al. 2011), *Drosophila* germline perinuclear nuage (Tejas and Tapas; Patil et al. 2014), and *Danio rerio* germ cell granular structures (TDRD7; Strasser et al. 2008; D’Orazio et al. 2020).

The presence of the LOTUS domain in a protein might be a good starting point to characterize its functions within the germline and to try to predict Oskar-like germ plasm or germ granule assembly factors in other species that lack an identified master regulator, i.e. a factor that is necessary and sufficient for the assembly. In our study, we approached the question in the bivalve *Ruditapes philippinarum*, an interesting developmental model. Besides an unusual modality of cytoplasmic inheritance known as Doubly Uniparental Inheritance (DUI) of mtDNA (Zouros et al. 1994; Milani et al. 2011) that makes it a unique and evolutionary stable study system for mitochondrial biology and inheritance, heteroplasmy, mitonuclear coevolution and genomic conflicts (Breton et al. 2007; Milani and Ghiselli 2015; Ladoukakis and Zouros 2017), *R. philippinarum* shares with other bivalves the annual renewal of gonads (Gosling 2003). Indeed, *R. philippinarum* gonads form every year at the beginning of the mating season. The gametogenic phase consists in the multistep differentiation of germ cells inside sack-like structures, called acini, and leads to the ripening of the gonad. During this phase, the gonadic tissue is located inside the connective tissue, near the intestine, and consists of acini that grow in dimension with the progress of gametogenesis (Devauchelle 1990; Gosling 2003; Milani et al. 2011). After the spawning period, clams are characterized by sexual rest, gonads are degraded, and sexes are no more recognizable.

The annual gonadic renewal appears to be preceded by proliferation in the intestinal epithelium of undifferentiated cells that express germline markers, like Vasph, the *R. philippinarum* Vasa orthologue (Milani et al. 2015, 2018). Similar Vasa-tagged intestinal cell clusters were observed also in other three bivalve species, comprising two species owing to another family, i.e. *Anadara kagoshimensis* and *Crassostrea gigas* (Pteriomorphia), suggesting a shared pattern for the whole class (Milani et al. 2017). However, a recent study of Cherif-Feildel and colleagues (2019) failed to observe labelling of the Vasa homologue in both the intestinal and the connective tissues of *C. gigas* but observed Vasa exclusively in early germ cells localized in the gonads. Moreover, in the germinal epithelium, the authors observed Vasa-tagged potential germ stem cells surrounded by what appeared to be a germinal niche of specialized somatic cells. It is clear how the characterization of the early germline stages in bivalves needs additional investigation, and the extensive diversity of the class represents a stimulating resource. Nevertheless, the annual renewal of the gonads is a shared characteristic and it would be interesting to understand how germ granules are initially segregated into the germline and how germline continuity is preserved by some specific cells during the non-reproductive season.

Despite germline determination mechanisms in clams are far from being understood, recent analyses showed the presence of germ plasm-related granules in the *R. philippinarum* germline (Reunov et al. 2019). In that work, such granules, that include Vasa-positive substance, have been observed through electron microscopy to arise at least twice during oogenesis: once right after the prezygotene/pachytene meiotic stage (these present also in the male lineage), and once in the late oocyte. These latter granules have been proposed to be selectively inherited in the germline lineage of the offspring, therefore determining it in a “preformation” mode of germline determination (Milani et al. 2018; Reunov et al. 2019). Indeed, early germ cells of both sexes show the presence of Vasa-tagged germ granules that dissolve during the differentiation of the lineage and, in concert with mitochondria, appear to induce the mitosis–meiosis transition of spermatogonia and oogonia (Reunov et al. 2019).

The aim of this study is to provide a better characterization of the germline formation in *R. philippinarum*. In this work, we explored the dynamics of germline development by in silico identification and in situ localisation of a newly identified germline marker for *R. philippinarum*. In details, starting from bioinformatic analyses on RNA-Seq transcriptomic data, we found a candidate possibly involved in the first stages of germline differentiation in *R. philippinarum* (TDRD7 homologue). We confirmed the in silico assembled sequence by Sanger sequencing, and we designed specific antibodies to target the protein in vivo. Immunohistochemistry and immunofluorescence assays were used to study the distribution of TDRD7 within histological samples containing gonadal tissue. These experiments were performed on male and female individuals collected during the reproductive season and during the sexual rest.

## Methods

### Sequence identification and analyses

To look for potential germ plasm master regulators in the Manila clam *R. philippinarum*, we started by BLASTing (Camacho et al. 2009) the *Drosophila melanogaster* Oskar protein sequence against the publicly available annotated bivalve proteomes (on the NCBI non-redundant protein database, or nr; taxid: 6544). We then used the best and only hit (*Mizuhopecten yessoensis* TDRD7A-like protein, accession code: XP_021379223.1) to look for the orthologue in *R. philippinarum* by BLASTing it against our de novo transcriptome. The *R. philippinarum* best hit was then back-BLASTed against *M. yessoensis* proteome (on nr; taxid: 6573) to assess homology (them being reciprocal best hits). The *R. philippinarum* transcriptome we used was built with Trinity v2.9.0 (Haas et al. 2013) on reads from gonads and somatic tissues (abductor muscle and mantle) of 8 female and 8 male samples, and consisted of 553,711 transcripts (N50: 1,337; high number of transcripts is likely due to samples polymorphisms). The transcriptomic samples were part of a transcriptomic profile analyses of *R. philippinarum* under review (NCBI BioProject Acc. No. PRJNA672267): total RNA was extracted with TRIzol, poly-A transcripts were isolated with magnetic beads and used as template for cDNA synthesis; the selected insert size was approximately 500 bp, and sequencing was performed on an Illumina HiSeq 2500 platform to generate 150 bp paired-end reads. Reads were previously trimmed with Trimmomatic-0.39 (Bolger et al. 2014) with the following parameters: LEADING:3 TRAILING:3 SLIDINGWINDOW:28:28 MINLEN:98, leaving approximately 171 million reads for Trinity v2.9.0 in silico assembly. Transcriptomic completeness was assessed with BUSCO through the gVolante online interface (percentage of complete core orthologues: 99.8%; https://gvolante.riken.jp/analysis.html).

Once we obtained the *R. philippinarum* orthologue transcript of the *M. yessoensis* TDRD7A-like protein, we extracted the translated coding sequence with NCBI ORFinder (https://www.ncbi.nlm.nih.gov/orffinder/). The sequence was then characterized for domain composition (with InterProScan; Jones et al. 2014). On the nucleotide sequence of the whole transcript, we designed 13 couples of PCR primers that covered the whole coding sequence in 9 overlapping sections (predicted with Primer3; Untergrasser et al. 2012; Supplementary Table 1). We used the primers to amplify the transcript portions and Sanger-sequence them to verify the existence of the whole transcript in vivo, therefore excluding the possibility of it being a de novo assembly construct. Samples for amplification came from two female gonadic samples of *R. philippinarum* (RNA extraction by TRIzol from fresh tissues, Thermo Fisher Scientific; retrotranscription with SuperScriptIV, Thermo Fisher Scientific; PCR cycles in Supplementary Table 1).

Moreover, since we obtained the protein sequence from a transcriptome of pooled specimens, we checked for the presence of the transcript in different samples and tissues by building different de novo transcriptomes for gonadic and somatic (abductor muscle and mantle) tissues of 8 females and 8 males (for a total of 32 transcriptomes; see before for assembly procedure and reads provenience). We also calculated and compared the levels of transcription of the *tdrd7* transcripts throughout the samples (with DESeq2, following the Trinity pipeline for Differential Expression Analysis; Love et al. 2014) and compared them with the germline marker *vasph* (*R. philippinarum* orthologue of the ubiquitous *vasa*; NCBI accession code: JO110167.1).

### Sampling

We performed histochemical analyses on specimens of the Manila clam *R. philippinarum* from Sacca di Goro (Adriatic Sea, FE, Italy), sampled in the gametogenic stage (May–July), and in the reproductive spent phase (November). The sex of gametogenic clams was determined via gametic smear observation under an optical microscope. The direct observation of gametes also allowed us to access the reproductive stage of the samples that can be only hypothesized before the sampling due to possible significant yearly environmental variations. Whole gonads and parts of the digestive tube were either dissected and directly processed for Immunohistochemistry (IHC) and Immunofluorescence (IF) or stored at − 80 °C for Western Blot (WB) analysis. In clams sampled during the reproductive spent phase (November), the entire body was dissected due to their tiny size and the difficulties of determining their sex. The number of samples used for each technique is specified in the dedicated paragraphs.

### Antibody production

We decided to investigate the factor in living tissues in the form of protein sequence (rather than mRNA localisation) because we were interested in the stages of actual expression of the factor function, and data would have been more directly comparable with previous works on Vasph protein detection in the same tissues (Milani et al. 2017, 2018). We utilized specific antisera produced in chicken by Davids Biotechnologie (Regensburg, Germany) to visualize *R. philippinarum* TDRD7 protein. These antibodies were generated against two synthetic peptides: the first one was synthesized from the first of the two predicted LOTUS domains present in the TDRD7 protein (peptide EKFILSMPDVARIDRRGGD, acronym EKF), while the other was synthesized from the second of the three predicted TUDOR domains (peptide AYDDGLYHRVRVMSVQDGKK, acronym AYD). The peptides were chosen among those with better score for epitope prediction (algorithm by Davids Biotechnologie). Moreover, we evaluated the position of the suggested peptides in the 3D structure predicted on the I-TASSER server (https://zhanglab.ccmb.med.umich.edu/I-TASSER/; Yang and Zhang 2015) and we chose external and easily reachable targets. The obtained antibodies were tested for immunoreactivity by ELISA with the immunogen peptides and were later purified by affinity chromatography (Davids Biotechnologie). Davids Biotecnologie also provided the synthetic peptides which were used for the primary antibodies production and that we used to test antibody specificity in the Western Blot assays.

### Immunolocalization

Females and males of *R. philippinarum* were analysed at two stages of the reproductive cycle (gametogenic and spent phase) to identify the localisation of TDRD7 protein in several tissues and cell types. The histological districts observed included germline (acini in gametogenic individuals) and somatic tissues (intestinal epithelium and connective tissue). Samples were processed with immunohistochemistry (IHC) and immunofluorescent (IF) protocols. For IHC staining, the entire body was processed following the method in Lazzari et al. (2014), while tissue samples for IF were processed as described in Milani et al. (2015).

For the IHC staining, consecutive sections from the same animal were incubated with the two anti-TDRD7 antibodies to compare their immunostaining pattern in adjacent histological layers. Sections were incubated with primary antibodies against TDRD7 (polyclonal anti-EKF or anti-AYD developed in chicken) diluted 1:100 and then with the secondary antibody HRP anti-chicken in goat (Santa Cruz Biotechnology Inc.) at the dilution of 1:100. Other sections were incubated with anti-VASA/VAS antibody (Abcam ab209710; polyclonal anti-Vasa developed in rabbit), diluted 1:100, and the secondary antibody HRP anti-rabbit in goat (Santa Cruz Biotechnology Inc.) at the dilution of 1:100. Negative controls for the specificity of immunostaining were obtained by omission of the primary antibodies, replaced by 3% normal goat serum. In the IF protocol, sections were incubated with the primary antibodies anti-EKF or anti-AYD diluted 1:1000 and the secondary antibody polyclonal goat anti-Chicken Dylight® 550 Cross-Adsorbed (Thermo Fisher) diluted 1:800. Negative controls for the specificity of immunostaining were obtained by omission of the primary antibodies, replaced by 1% normal goat serum and 3% bovine serum albumin. Given that we observed coincident immuno-profiles for both IHC and IF for the two antibodies, we will subsequently refer in general to anti-TDRD7 staining.

IHC imaging was performed on a total of 25 sections from 9 specimens (3 gametogenic males, 3 gametogenic females, and 3 individuals in spent phase) with Olympus BH-2 microscope (Olympus S Plan Achromatic objective 10×, numerical aperture 0.30, working distance 7.50 mm, focal length 18.98 mm, and Olympus S Plan Achromatic objective 20×, numerical aperture 0.46, working distance 1.50 mm, focal length 8.03 mm; both with Tube length/coverslip thickness 160/0.17 mm). Images were acquired with BEL Photonics BlackL 5000 USB digital camera (5 Mpixel) through the acquisition software BEL Photonics Eurisko 2.9 (auto exposure, 8 bit RGB images recorded in TIFF format, 14.2 MB in size, pixel dimensions: 2592 × 1920). Images of IF staining were acquired by confocal laser scanning microscope (Leica confocal SP2 microscope; Leica Microscope Objective HCX PL APO 63×/1.32–0.6 Oil CS; image dimension: 1140 × 968 pixels) using Leica software with a total number of 100 sections from 10 specimens (4 gametogenic males, 4 gametogenic females, 2 individuals in spent phase). Fluorophores used were DyLight® 550 for the secondary antibody (Ex: 562 nm, Em: 576 nm, Gain 825 V, offset 0) and TO-PRO-3 for nucleic acids (Ex: 642 nm, Em: 661 nm, Gain 578 V, offset 0).

### Western blot

WB was carried out following the method in Milani et al. (2015). Gametogenic male and female clams from early and late July were used to obtain gonadic homogenates for WB (per sex: 2 samples in early July and 3 samples in late July; loading 20–40 µg of total protein homogenate per lane). Both primary antibodies against TDRD7 (anti-EKF and anti-AYD) were diluted 1:1000, anti-VASA was diluted 1:2000, while secondary antibodies conjugated with the horseradish peroxidase (HRP anti-chicken or anti-rabbit in goat, respectively; Santa Cruz Biotechnology Inc.) were diluted 1:5000. To monitor the antibody specificity, synthetic peptides were incubated for 30 min with the primary antibody solution at a 20-fold excess concentration before use.

## Results

### Sequence identification

The Oskar (short isoform, i.e. the one that promotes germ plasm assembly) BLASTP against Bivalvia subset on NCBI nr database gave as best output hit *Mizuhopecten yessoensis* TDRD7A-like protein (XP_021379223.1; bitscore: 48.9; e-value: 6e-05; identity percentage of alignment region: 30.23%). The two proteins aligned exclusively in the amino acid positions 18–98 on the Oskar sequence that coincided with the LOTUS domain (Oskar only LOTUS: position 14–83 as inferred by Interproscan-PROSITE; TDRD7A-like first LOTUS out of two: positions 3–76 as inferred by Interproscan-PROSITE). Indeed, Oskar has no homologues outside the Insecta lineage, but nevertheless shares the presence of the LOTUS domain with other Tudor-family proteins (see Introduction). The *R. philippinarum* pooled transcriptome showed 5 transcript isoforms (1 did not cover the whole ORF but represented a truncated 5′ transcript rather than a length isoform) that positively aligned with high-quality values against *M. yessoensis* TDRD7A-like (best TBLASTX isoform hit: bitscore 288; e-value 1.72e−81; identity percentage 33.555%). These transcripts had almost identical sequences between each other and we considered them as between-individual polymorphisms (16 specimens were pooled to build the reference transcriptome), rather than actual biological isoforms: three transcripts had 100% amino acid identity (comprising the truncated one) and this sequence was uploaded on GenBank (MW170385), one had a single amino acid substitution, and one had an insertion of a single amino acid. This was confirmed by transcriptomes of single individuals that did not show sequence isoforms within them. Therefore, we could assess the presence of the TDRD7 orthologue (confirmed by a reverse BLAST against *M. yessoensis* that obtained as best hit the starting protein) in single copy in the *R. philippinarum* transcriptome. Moreover, the Sanger sequencing of 9 overlapping regions that comprehended the whole ORF confirmed the presence of the whole coding region of the transcript in vivo.

**Fig. 1:**
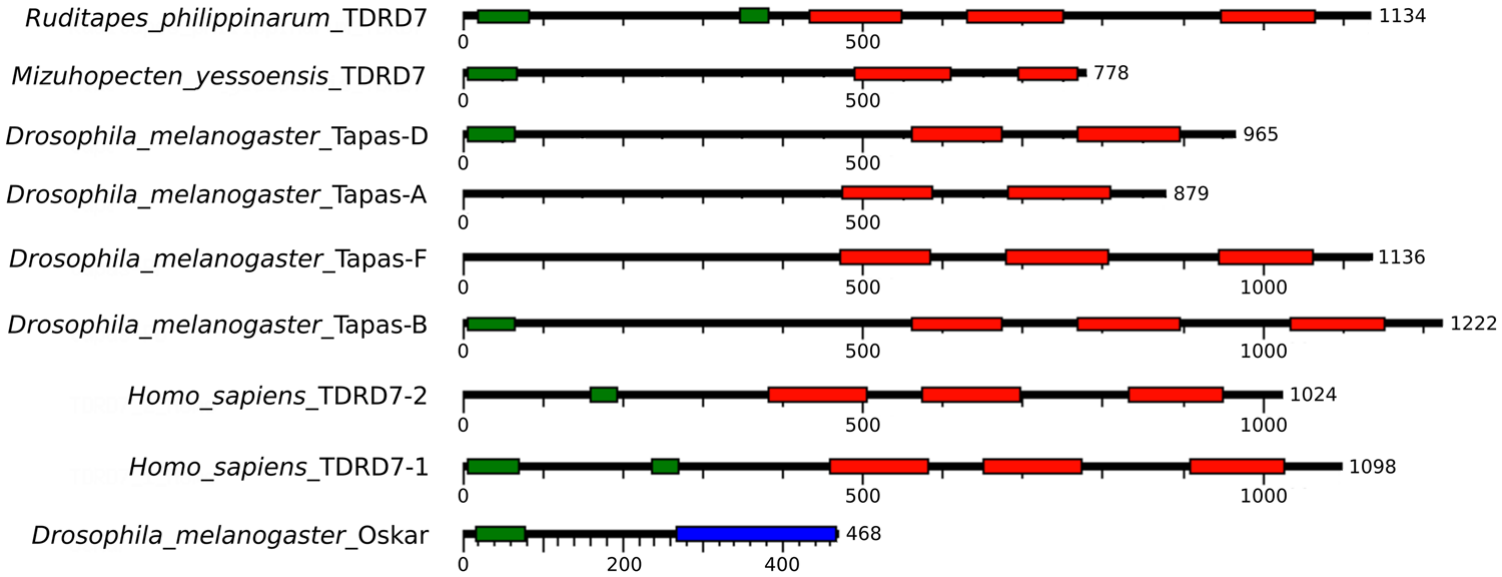
Domain composition of TDRD7 homologues in different species and *Drosophila* Oskar. LOTUS (green) and TUDOR (red) domains are highlighted. OSKAR domain of Oskar is depicted in blue. For *D. melanogaster* and *Homo sapiens* all known isoforms are shown for comparison with *Ruditapes philippinarum* and *Mizuhopecten yessoensis* domain composition

The transcript included an ORF of 1134 amino acids (GenBank accession number MW170385; predicted molecular weight: 126.8 kDa), with 5 annotated domains (Fig. 1): two LOTUS domains in amino acid positions 20–92 and 361–429 (PROSITE database in InterProScan); three consecutive TUDOR domains in the amino acid positions 447–563, 644–764, and 962–1078 (Pfam database in InterProScan).

### Within individual in silico transcription of TDRD7

A single transcript comprising the whole coding sequence of *tdrd7* was not always present in single-individual transcriptomes. However, the presence/absence and the level of completeness is itself a signal: we will use the term “complete” when the coding sequence is comprehended within a single transcript; “split” when the coding sequence is complete but fragmented in 2 or 3 transcripts; “incomplete” when the coding sequence is either incomplete or fragmented in many short transcripts. Among the 8 female gonad transcriptomes, *tdrd7* was complete in 3 individuals, split in 2, and incomplete in 3. However, in male samples, only a single individual displayed a complete transcript of *tdrd7*, while 2 samples were split, and 5 were incomplete. Moreover, out of the 16 somatic transcriptomes, only one sample was complete (a female), 3 were split (two males and a female), and 12 were incomplete. On the other hand, *vasph* was present and complete in 30 out of the 32 transcriptomes (16 gonadic and 16 somatic; the two incomplete ones, for only a few amino acids, were somatic samples).

*Tdrd7* was differentially transcribed between tissues, with a moderate but highly significant upregulation in the gonads (~ 2.5 times more transcribed considering pooled sexes; *p*-value = 1.4e−12). On the other hand, the conserved germline marker *vasph* was transcribed ~ 8.5 times more in gonads than in somatic tissues (*p*-value = 3.3e−24; pooled sexes). The difference between *tdrd7* and *vasph* lied at the level of gonadic transcription, that was almost double for the latter (average of normalized counts: 225.8 for *tdrd7* and 515.2 for *vasph*), rather than somatic transcription (88.6 against 60.7, respectively).

Considering sexes separately, the differential transcription significance held for both transcripts (always upregulated in gonads), but the intensity of the signal was stronger in females (*tdrd7* ~ 3.1 times more transcribed; *vasph* ~ 11 times more transcribed) than in males (*tdrd7* ~ 2.1 times more transcribed; *vasph* ~ 5.5 times more transcribed). However, such differences were mostly confined to the transcriptional level of *vasph* that was almost 3 times more transcribed in female gonads than in male gonads. Indeed, the transcription of *tdrd7* was statistically equal between the sexes. Lastly, the transcriptional level of the two genes in the somatic tissues were low and equal between the two sexes.

### Histological organization of the *R. philippinarum* gonad during the two stages of the reproductive cycle

**Fig. 2:**
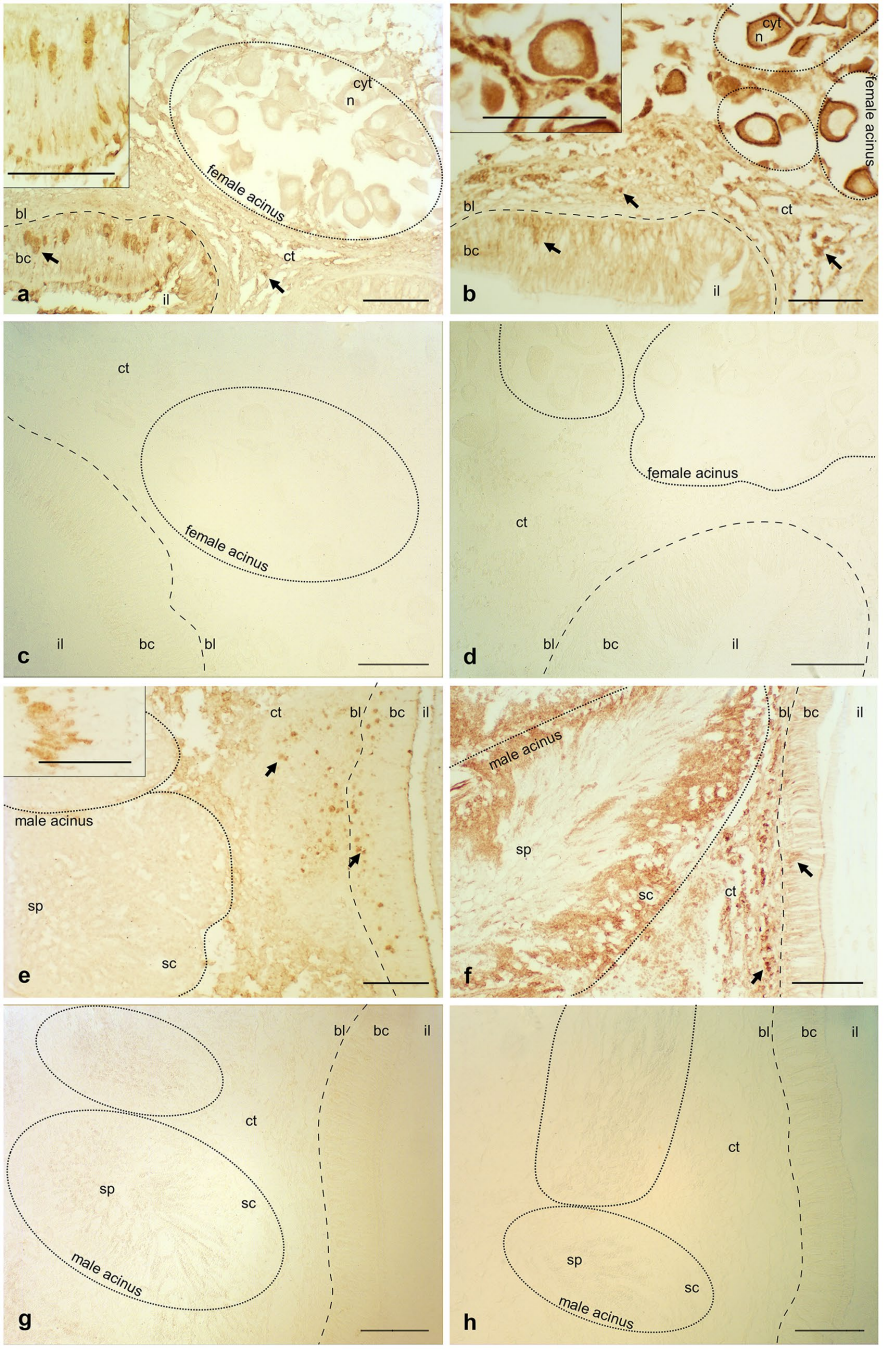
*Ruditapes philippinarum* tissues in gametogenic phase stained with immunolocalization of TDRD7 and Vasa. Left column: TDRD7 immunostaining and controls; right column: Vasa immunostaining and controls. **a** Female section stained with anti-TDRD7; **b** Female section stained with anti-Vasa; **c** Female control section with omission of primary anti-TDRD7 antibody (only secondary antibody HRP anti-chicken in goat); **d** Female control section with omission of primary anti-Vasa antibody (only secondary antibody HRP anti-rabbit in goat); **e** Male section stained with anti-TDRD7; **f** Male section stained with anti-Vasa; **g** Male control section with omission of primary anti-TDRD7 antibody (only HRP anti-chicken in goat); **h** Male control section with omission of primary anti-Vasa antibody (only secondary antibody HRP anti-rabbit in goat). In both female and male samples, TDRD7-labeled cells were present in the thickness of intestinal epithelium (arrows), between unstained columnar, batiprismatic cells (bc) close to the basal lamina (bl) (clusters of stained cells are magnified in the insets in **a** and **e**) and in the connective tissue (ct). No anti-TDRD7 staining was visible in the cytoplasm (cyt) and nuclei (n) of oocytes (**a**) or in spermatozoa (sp) (**e**). In both sexes, anti-Vasa staining was present in cells localized in the thickness of intestinal epithelium (arrows) close to the basal lamina (bl) and in cells inside the connective tissue (**b** and **f**). In female acini, anti-Vasa staining was the strongest in cells around the acinus wall (corresponding to earlier germ cells; see inset in **b**), and it was also present in mature oocyte cytoplasm (cyt), more concentrated at the cortex (c). In male acini, anti-Vasa staining was the strongest in cells at the acinus wall (corresponding to spermatogonia, spermatocytes and, apparently, up to early spermatids; see **f**), but it was not visible in spermatozoa (sp) inside the acinus lumen. Intestinal lumen (il). No staining was detected when the primary antibodies were omitted (**c**, **d**, **g**, and **h**). Brown: anti-TDRD7 and anti-Vasa staining. Scale bars = 100 µm

During the gametogenic phase, in all sections, intestinal epithelium and acini full of developing gametes were present (Fig. 2). The intestinal epithelium was 100–200 μm thick and it consisted in a columnar single-cell layer lying on a basal lamina. Beneath it, connective tissue surrounded the gametogenic acini that constitute the gonads and separated them from the basal lamina.

**Fig. 3:**
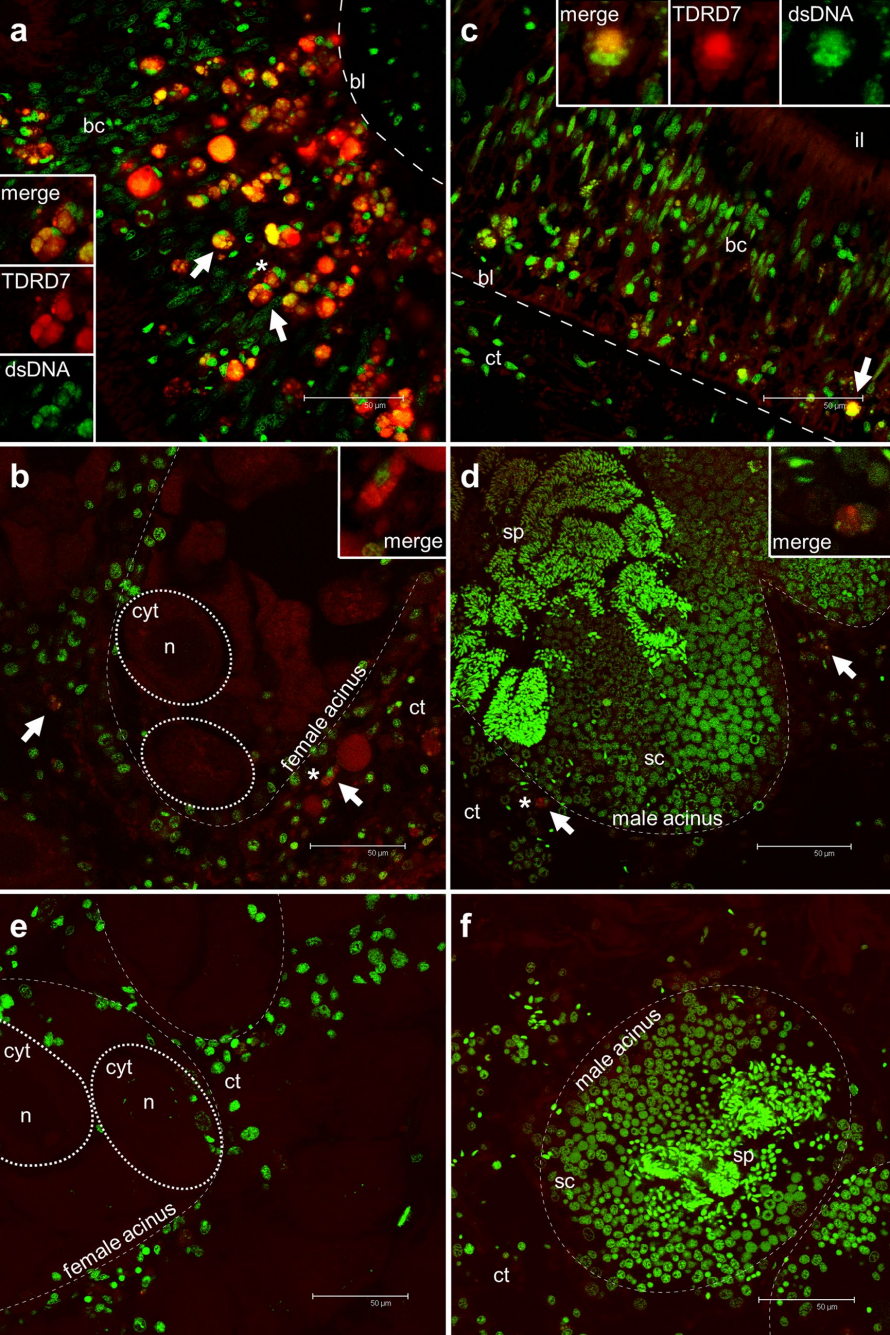
Immunolocalization of TDRD7 in germ cells of gametogenic females and males by IF assays. **a** Portion of intestine in the female section where many cells are stained with anti-TDRD7. In particular, anti-TDRD7 highlighted many putative PriSCs localised in the thickness of intestinal epithelium (arrows), between unstained batiprismatic cells (bc), close the basal lamina (bl). Some cell clusters (asterisk) are magnified in the insets. **b** The connective tissue (ct) surrounds mature female acini (dashed line). Some germ cells labelled with anti-TDRD7 surround acini full of eggs (arrows); two oocytes are highlighted with a dashed oval: oocyte cytoplasm (cyt), oocyte nucleus (n) (*cell magnified in the inset). **c** Anti-TDRD7 staining in a portion of intestine epithelium in male section where, like in female intestine portions, the primary antibody labels many germ cells (arrows) (*cell magnified in the inset); **d** Portion of a male acinus (dashed line) full of spermatocytes (sc) and spermatozoa (sp). Only few cells (arrows) in the surrounding connective tissue are stained with anti-TDRD7 (*cell magnified in the inset). **e** Female control section, and **f** Male control section treated exclusively with the anti-chicken secondary antibody (DyLight 550): no staining was detected. Red: anti-TDRD7 staining; Green: TO-PRO-3 nuclear dye. Scale bars = 50 µm

In female samples, acini appeared full of oocytes (Fig. 2b). The large nucleus of the oocytes was clearly visible. GCs in different meiotic phases were distinguished by their size and by nuclear chromatin morphology through fluorescent nuclear staining (Fig. 3). Early vitellogenic oocytes were oval in shape, while mature oocytes were roundish, and measured up to 80 μm in diameter (Fig. 3b).

Male acini (Fig. 2e, f) were in general more compact and closer together compared to female acini. There was a clear centripetal organization of spermatogenesis within each acinus, that was more clearly recognizable in IF images (Fig. 3d, f). From the periphery to the acinus lumen, spermatocytes, spermatids, and spermatozoa were recognizable by the different nuclear staining concentration and shape. The round and scarcely condensed nucleus of spermatocytes became tightly compressed and elongated in shape in spermatozoa.

Some samples were collected in the winter, during the spent phase, and in these sections, no acinus with gametes was present, as expected (see Supplementary Fig. 1).

### TDRD7 detection by immunohistochemistry (IHC) assay

During the gametogenic phase, in the intestinal epithelium, TDRD7 antibodies stained only some cells located near the basal lamina and dispersed between the unlabelled batiprismatic cells that form the intestinal epithelium (Fig. 2a, e). The immunostaining was concentrated in almond-shaped structures about 20 μm in length or smaller (Fig. 2a, e). No TDRD7 staining was visible in either oocytes or spermatozoa, while slight staining was present in cells in the connective tissue close to the intestinal epithelium. In individuals in the spent phase, anti-TDRD7 staining was very faint in the same type of cells, in the intestinal epithelium and in the connective tissue (Supplementary Fig. 1).

Vasa staining was visible in the same type of cells localized in the intestinal epithelium and inside the connective tissue (Fig. 2b, f). In the case of Vasa staining, the labelling was the strongest in the cells in the connective tissue and inside the acini, both in females and males, while it appeared weaker, although present, in the cells inside the intestinal epithelium (Fig. 2b, f). In female acini, the staining was stronger at the periphery, where earlier germ cells are present. Mature oocytes in the acinus lumen were characterized by the presence of granules labelled by anti-Vasa in the cytoplasm, more concentrated in the cortical region of the oocytes (Fig. 2b). In male acini, anti-Vasa staining was stronger in the peripheral area as well, where germ cells at different stages of differentiation are localized, while mature spermatozoa accumulated in the acinus lumen appeared not stained (Fig. 2f).

Negative control sections, in which the primary antibody was omitted, showed no staining over all histological structures (Fig. 2c, d, g, h; Supplementary Fig. 2).

### TDRD7 detection by immunofluorescent (IF) assay

Immunofluorescence images at the confocal microscope helped to appreciate the localisation of TDRD7 within single cells thanks to higher resolution and contemporary nuclear staining. Many anti-TDRD7 labelled cells were localised within the intestinal epithelium and were significantly different in shape from unstained columnar, batiprismatic cells (Fig. 3a, c): they had a round nucleus and were often positioned close to the basal lamina. Some of these cells were clustered in small groups of 2–3 close to each other and were strongly stained with anti-TDRD7 antibodies (Fig. 3a, c insets), clusters that likely correspond to the almond-shaped stained structures seen with IHC. In these clusters, one cell appeared exclusively labelled with anti-TDRD7 in its cytoplasm, no anti-TDRD7 staining was visible in the nucleus, in which only the nuclear dye was detected; the remaining cells were also marked with the anti-TDRD7 antibodies in their cytoplasm, but the staining was also present in the nucleus, and the overlapping was shown as yellow fluorescence in the image with merged channels. This was observed in both male and female specimens (Fig. 3a, c insets).

Around the female acini (Fig. 3b), some cells were slightly stained by anti-TDRD7 (Fig. 3b inset). Oocytes showed a diffuse and slight anti-TDRD7 staining in the cytoplasm, while anti-TDRD7 staining in the nucleus was not visible. In males (Fig. 3d), only few cells outside the acini showed a strong anti-TDRD7 staining. The nucleus of these cells presented lightly packed chromatin, and anti-TDRD7 staining seemed more intense at one side of the nucleus (Fig. 3d inset). No evident anti-TDRD7 labelling was detected in the cells in the intestinal epithelium of adults sampled during the spent phase of reproductive cycle (see Supplementary Fig. 1b).

Controls on male and female sections treated exclusively with secondary antibody showed no labelling in any histological structure (Fig. 3e, f;).

### Western blot

**Fig. 4:**
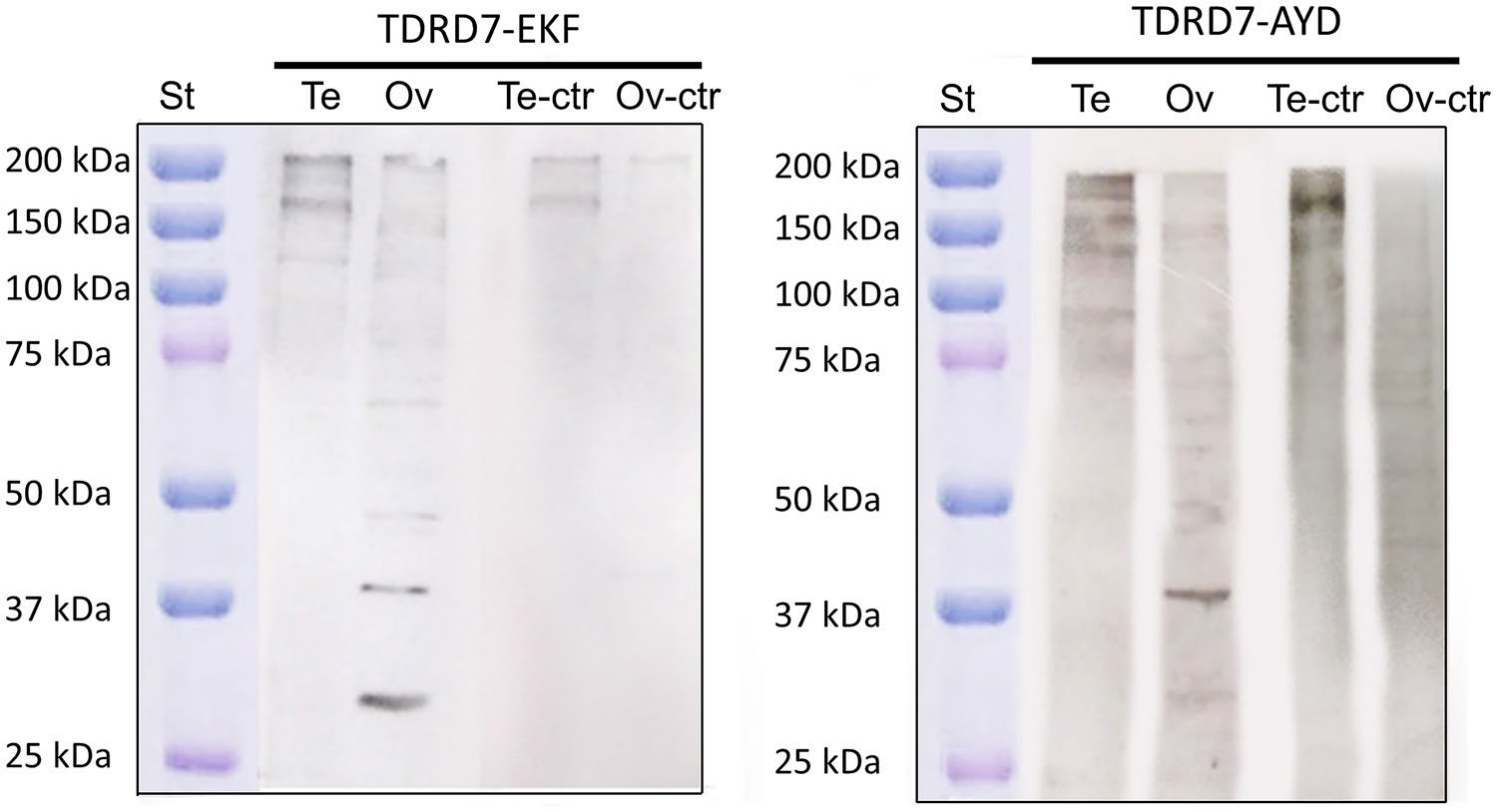
Immunoblot analysis with anti-TDRD7 antibodies on testis and ovary extracts of adult *Ruditapes philippinarum* (WB example from late July clams corresponding to samples M4 and F4 in Supplementary Table 2). Anti-TDRD7-EKF (left panel) and anti-TDRD7-AYD (right panel). In female extracts (Ov), both antibodies displayed bands at 30, 37, 50, 70 kDa, but with different intensity of the staining. Few close bands at high molecular weight were detected in male extracts (Te). One of these, for anti-TDRD7-EKF, even if faint, was close to the weight of the predicted protein (126.8 kDa) and disappeared with the control peptide incubation. A clear reduction of the band intensity is visible in controls (Te-ctr and Ov-ctr). The molecular weight of the protein standard (St; Precision Plus Protein™ Dual Color Standard Bio-Rad) is reported in kDa on the left side of each panel. See also Supplementary Table 2 for WB result variability

Western blot was performed on male and female gonad homogenates of *R. philippinarum* using both produced antisera (anti-EKF and anti-AYD antibodies; Supplementary Table 2; Fig. 4) and anti-Vasa (Supplementary Fig. 3). This analysis was not performed on animals sampled in reproductive spent phase due to the absence of gonads in that stage of the reproductive cycle.

The blot profiles were not always concordant between both sexes and individuals, and the number and weight of the bands were variable. Usually anti-AYD profiles displayed more bands than anti-EKF ones. One band that was present in almost all specimens (in 4 out of 5 females and 2 out of 5 males) with both antibodies was approximately 37 kDa (Fig. 4; Supplementary Table 2). Other bands of 30, 50, 60 and 70 kDa were displayed in different blot profiles. Bands around the predicted molecular weight of the translated transcript of *tdrd7* (126.8 kDa) were observed for both antibodies in both sexes, but they were not always present.

To test the specificity of antisera, the antibodies were pre-incubated with a 20-fold molar excess of the peptides against which they were produced. This step was performed to chelate by competition every antigenic site of the primary antibody. Both controls showed significant reduction of some band intensity, suggesting good specificity of antibodies. Bands around 130 kDa completely disappeared with the peptide incubation.

## Discussion

### A candidate protein for early stages of germline differentiation

We tried to identify factors that could act in the early phases of germline specification in *R. philippinarum* that could possibly share functions as assemblers of germ granules with Oskar of *D. melanogaster*. It has been observed how, in *R. philippinarum*, some germ plasm-related structures are present in germ cells at initial stages of differentiation (Reunov et al. 2019). Since these germ plasm granules are present at early germline stages in both sexes, the presence of a scaffolding protein, if not a germ plasm master regulator, would seem plausible in clams and maybe in bivalves in general. This putative protein, or proteins, would be able to establish germline fate in male and female annual gonad formation and possibly recruit Vasph, either directly or indirectly through germ plasm assembly. Recent studies on Oskar functional domains provided new models by which Oskar could promote germ plasm assembly by interaction between its LOTUS domains and Vasa (Jeske et al. 2015, 2017). For this reason, we focused on the presence of Oskar-like proteins, or proteins containing homologous domains, in *R. philippinarum* transcriptome.

We performed a BLAST search of Oskar against publicly available sequences of bivalves*,* finding no orthologues sequences, as expected. However, the only hit we found was the TDRD7 orthologue of *Mizuhopecten yessoensis*. The alignment similarity was confined to the LOTUS domain that this protein shares with Oskar. We then annotated through BLAST the TDRD7 orthologue in our *R. philippinarum* transcriptome and observed that the gene was differentially transcribed in gonads (up to 3.1 times more transcribed respect to somatic tissues in females).

In Metazoa, TDRD7 orthologs show conserved structural organization and present both TUDOR and LOTUS domains (Fierro-Constaín et al. 2017). TUDOR is thought to act as a scaffold for localisation of other nuage components (Thomson and Lasko 2005; Hosokawa et al. 2007), while LOTUS is involved in dimerization and in the recruitment of Vasa (Jeske et al. 2017). With InterProScan (Jones et al. 2014) we inferred the domain composition of *R. philippinarum* TDRD7 (Fig. 1): two LOTUS domains were present toward the N-terminal region of the protein and three consecutive TUDOR domains were present toward the C-terminal region. The conservation of TDRD7 proteins in Metazoa (Patil et al. 2014; Fierro-Constaín et al. 2017), the role of TUDOR and LOTUS domains within the germ plasm (Shukalyuk and Isaeva 2012), and the involvement of TDRD7 homologues in the assembly of germ granules in other species (Strasser et al. 2008; Tanaka et al. 2011; Patil et al. 2014; D’Orazio et al. 2020) allowed us to consider a TDRD7 orthologue as a worthy investigative unit for the study of germline specification and germ plasm assembly in *R. philippinarum*.

Indeed, the Tudor protein family is one of the most conserved groups of molecular determinants in germline specification (Fierro-Constaín et al. 2017). The TUDOR domain is commonly found in a wide range of proteins that are involved in RNA metabolism and splicing, histone modification, DNA damage response, cell division, differentiation, genome stability and gametogenesis (Hosokawa et al. 2007; Skorokhod et al. 2011; Pek et al. 2012). These determinants are usually organized in ribonucleoprotein complexes in the germ plasm of PGCs and/or nuage localised near nuclear pores (Fierro-Constaín et al. 2017). TUDOR domain-containing proteins also localise in germline-related cytoplasmic structures, such as the intermitochondrial cement of spermatocytes and the chromatoid bodies of spermatids in *Drosophila* and mice (Chuma et al. 2006; Handler et al. 2011; Yabuta et al. 2011). Interestingly, it has been observed how homologues of TDRD5 and TDRD7 are key factors for the biogenesis and assembly of germ plasm-related structures of different species (mouse, fruit fly, and zebrafish), them being disorganized in their absence (Strasser et al. 2008; Tanaka et al. 2011; Yabuta et al. 2011; Patil et al. 2014; D’Orazio et al. 2020). Such proteins include both TUDOR and LOTUS domains, and the concerted functions of protein recruitment (TUDOR domain; Thomson and Lasko 2005; Hosokawa et al. 2007Kirino et al. 2010), dimerization and Vasa interaction (LOTUS domain; Hosokawa et al. 2007; Patil et al. 2014; Jeske et al. 2015, 2017), make them interesting investigational units for the characterization of germline and germline-related cytoplasmic supramolecular structures, in whose assembly they can be possibly involved.

### TDRD7 is localised in early germ cells

Through immunolocalization experiments, female and male specimens of *R. philippinarum* were observed at two stages of development (gametogenic and spent phase) to identify the localisation of TDRD7 in tissues and cell types. The staining profiles of the two antibodies were perfectly consistent with each other: both antibodies labelled the same histological districts in both female and male samples. Nevertheless, differences existed between the staining localisation in gametogenic males and females, and specimens in sexual rest. In both male and female gametogenic phases, TDRD7 was localised in cells (1) in the intestinal epithelium (forming small clusters of few, closely associated cells), (2) in the connective tissue (also organized in small cell groups), and (3) in the gonadic acinus periphery, where the protein was located in germ cells probably at initial stages of gametogenesis, being comparable to those previously reported to express Vasph (Milani et al. 2015, 2018). These anti-TDRD7 labelled cells were characterized by a round nucleus with highly condensed chromatin. On the other hand, in the reproductive spent phase, the TDRD7 staining was consistently lighter but limited to the same cells described above in (1) and (2).

The annual renewal of gonads in bivalves is preceded by proliferation of Vasa-tagged cells within the intestinal epithelium (Milani et al. 2017, 2018; but see Cherif-Feildel et al. 2019). These cells were supposed to migrate later in the connective tissue, where they divide and eventually differentiate into germ cells and gametes inside newly formed acini. In the present study, the observed immunohistological patterns of TDRD7 support it as a candidate protein involved in the first stages of annual germline differentiation. We could identify with anti-TDRD7 tagging all germline-related cells that were previously characterized for *R. philippinarum* with Vasph (from undifferentiated clusters in the intestinal epithelium, to sparse cells immersed in the connective tissue, to developing acini at diverse differentiation stages; Milani et al. 2015, 2018) making it a strong evidence of the involvement of such protein in the germline differentiation pathway.

TDRD7 roles in this process are far to be clarified. In mice, it has been observed how the protein is crucial for the biogenesis and assembly of male germline chromatoid bodies (Tanaka et al. 2011). Interestingly, similar results were obtained also for TDRD7 paralog, the protein TDRD5 that was assessed as fundamental for the proper assembly of the same cytoplasmic structures (Yabuta et al. 2011). The fact that both proteins share the presence of TUDOR and LOTUS domains suggests that they might cover similar functions in quality/quantity of the assembly and scaffolding of germline determinants. Also in *Drosophila*, these two proteins have been observed in similar functions and districts: the insect Tejas (homologue of TDRD5) and Tapas (homologue of TDRD7) are crucial for the nuclear localisation of Piwi (Patil et al. 2014). Tapas interacts with Vasa and piRNA pathway proteins allowing their localisation in the nuage, a perinuclear supramolecular structure equivalent to mouse chromatoid bodies (Patil et al. 2014). Another model organism in which TDRD7 has been associated with proper formation of germ cell perinuclear granules is *Danio rerio*. Here, disruption of granule architecture was observed after TDRD7 loss-of-function (Strasser et al. 2008), and mis-localisation of germ plasm due to TDRD7 disruption led to somatic differentiation of PGCs (D’Orazio et al. 2020) Moreover, TDRD7 is also involved in cytoplasmic structures in the mouse embryonic ocular lens formation, i.e. in somatic tissues (Lachke et al. 2011). Here, the protein is involved in RNA recruitment and the formation of RNA granules (Lachke et al. 2011), suggesting that the control of ribonucleoprotein aggregates is the common mode of action of TDRD7 in different species and tissues.

In our species, the fact that TDRD7 is present since the very first stage of the annual gonad renewal (as supposed for the intestinal cell clusters) is evidence of its involvement in the early steps of germline differentiation; vice versa, given TDRD7 role in germline differentiation in many animals, its presence together with Vasph in such cells supports the involvement of intestinal cell clusters in germline establishment. TDRD7 high degree of concurrence with Vasph, and the presence of LOTUS domains in it (known to interact with Vasa), might mean that also physical interactions run among these two germline key factors (Jeske et al. 2015, 2017). However, additional histochemical tags comprising both proteins possibly by immuno-TEM analysis, as well as immunoprecipitation analyses, might shed light on the issue. It would be interesting to investigate the presence of TDRD7 in germ plasm-related Vasph-tagged granules that have been observed in *R. philippinarum* oocytes and PGCs and that have been proposed as being the units of selective inheritance for the putative preformation germline specification mode in clams (Reunov et al 2019).

It is possible that the *R. philippinarum* orthologue of TDRD7 is involved exclusively in the early stages of gametogenesis, as previously hypothesized in mouse and fruit fly (Hosokawa et al. 2007; Patil et al. 2014). Indeed, in female and male specimens, TDRD7-stained cells with round nucleus were localised around the acinus wall (Figs. 2a, e; 3b, d) and they likely represented cells at initial stages of gametogenesis, i.e. oogonia and spermatogonia, respectively. This hypothesis is also supported by anti-TDRD7 strongly stained cell clusters within the intestinal epithelium: the peculiar, clustered organization was present only in male and female specimens in the gametogenic phase, and not in the spent phase, where only a few slightly stained cells were present. Cell groups within intestinal epithelium has already been observed with anti-Vasph immunofluorescence (Milani et al. 2015, 2018) and interpreted as totipotent cells involved in the annual gonad renewal (we will refer to these cells as PriSCs to be in line with previous interpretation: whether they actually maintain somatic potential like intestinal regeneration, i.e. proper PriSCs, or only germ cell fate, that would make them PGCs, is still a matter of debate and more cytological analyses are needed). In respect to previous works with the staining of a different marker, here we could observe some sort of within-cluster differentiation: in each cluster, one of the cells did not seem to contain anti-TDRD7 staining in the nucleus, while the remainder cells did. All the cells appeared to contain TDRD7 in the cytoplasm. In other species, TDRD7 orthologues have been observed in cytoplasmic, mostly perinuclear positions. However, western blots of cellular fractions revealed the presence of a short isoform of human TDRD7 (60 kDa) in the nucleus, while longer isoforms were located in cytoplasmic and mitochondrial fractions (Skorokhod et al. 2011). Moreover, Tanaka and colleagues (2011) observed the presence of TDRD7 in nuclei of mammal intermediate differentiating germ cells, i.e. postnatal spermatogonia and early meiotic spermatocytes, while previous and subsequent differentiation stages contained the protein exclusively in the cytoplasm. These observations suggest that TDRD7 executes its functions in different cellular compartments, either by translocation or by selective isoform localisation. The TUDOR domains included in TDRD7 might be involved in the proper assembly of cytoplasmic granules, but they might also have other nuclear-related functions. Indeed, the TUDOR domain has also been associated in other proteins to histone binding and DNA damage response (Pek et al. 2012; Botuyan and Mer 2016); therefore, a direct nuclear role for TUDOR-containing proteins has been assessed, and it could be possible for TDRD7 to be involved with different functions in different cellular districts.

In our species, we could observe the protein in the nucleus exclusively within germ cells in early differentiative stages, i.e. in intestinal clusters. All free-roaming cells in the connective tissue, and some of the intestinal ones displayed only cytoplasmic tagging of anti-TDRD7. It could be possible that this different pattern can differentiate the daughter cell that keeps full PriSC totipotency from those cells that have already started their germline differentiation (possibly PGCs), that will later result in migration and colonisation of the connective tissue. The cells with TDRD7 in the nucleus might represent an intermediate stage between the inactive phases of totipotent cells and free-roaming germ cells in the connective tissue with established germline differentiation fate. Following this supposition, the translocation/localisation of TDRD7 in the nucleus would have roles in the genetic activation of germline fate, and later it would translocate/localise in the cytoplasm where it could be involved in other germline-related functions. If this was the case, then TDRD7, along with Vasph, would be expressed since the very first step of annual germline renewal, but TDRD7 presence would also allow to discriminate PriSC activation and split in one totipotent and one germline-related pluripotent cell.

### Possible alternative isoforms of TDRD7

Ambiguous observations resulted from the western blot analysis. In previous works, western blots of subcellular fractions (cytoplasm, mitochondria, nucleus; Skorokhod et al. 2011) and immunofluorescence (Hosokawa et al. 2007; Patil et al. 2014) allowed to investigate the localisation of TDRD7 in the mouse and the fruit fly. It was mostly found into specific structures in the cytoplasm: on the mitochondria outer membrane and in the perinuclear zone (Hosokawa et al. 2007; Skorokhod et al. 2011) and localised to the perinuclear nuage with Vasa (Patil et al. 2014). Four alternative TDRD7 isoforms were identified in different subcellular localisation in human HeLa cells (Skorokhod et al. 2011): higher molecular weight bands (160 kDa TDRD7α and 130 kDa TDRD7β) were found in the cytosolic fraction, while other protein bands of 110 kDa (TDRD7γ) and 60 kDa (TDRD7δ) were detected in mitochondrial and nuclear fractions, respectively (Skorokhod et al. 2011). Four isoforms have also been annotated in *Drosophila* (predicted molecular weights ranging from 99.7 to 138.1 kDa; https://flybase.org/download/sequence/FBgn0027529) and two in *Mus musculus* (predicted molecular weights of 122.2 and 125.9 kDa; ENSMUSG00000035517: Ensemble database).

Some alternative bands of TDRD7 also seem to be present in our animal system (Fig. 4; Supplementary Table 2) and this might suggest the existence of potential isoforms of TDRD7 in *R. philippinarum*. Unfortunately, our protocol consisted in the collection of the entire gonad and its subsequent homogenization, which consequently did not allow us to distinguish the different subcellular fractions as previously shown by other research groups (Skorokhod et al. 2011). Nevertheless, this was overcome by performing immunolocalization protocols. Since alternative isoforms of TDRD7 were already found in other model system, it is possible that this is a common feature of this gene family and the presence of isoforms might be compatible with different functions performed inside the cells (Patil et al. 2014; Skorokhod et al. 2011), maybe acting in different steps of GC differentiation and in the different sexes. However, in our species, the situation looks convoluted: males and females had different western blot profiles, and the bands that approximately corresponds to the predicted molecular weight of the protein (126.8 kDa) were not always present. The fact that isoforms at the transcript level were completely absent in the transcriptome built with 16 samples of both sexes makes the interpretation of western blot even more complicated. To this, it has to be added the fact that histochemical observations did not show significant differences neither between sexes, nor between different antibodies, thus they all appear to act in the same tissue districts. To shed light on the issue, genomic data and exons characterization might help, but also western blot band sequencing and plasmidial amplification of the protein could yield clarifying results. As for now, an explanation of the cryptic and heterogeneous western blot profiles might lie in the fact that *R. philippinarum* specimens are not always perfectly synchronized in their gametogenic phase. Even individuals sampled simultaneously from the same populations can be found at different moments of germ cell maturation. TDRD7 might have different isoforms that function in different districts and moments of germline differentiation (as immunofluorescence profiles of intestinal clusters suggest), and the between-individuals heterogeneity of reproductive phases in clams might partially explain the inconsistency of western blot profiles between samples. Nevertheless, the neat consistent signal of both immunofluorescence and immunohistochemistry analyses in different sexes and with different antibodies allow us to exclude high levels of non-specificity for the antibodies, also considering that multiple bands with unpredicted molecular weights are present even in commercially available antibodies build in and for human cells.

## Conclusion

With the present work, we suggest TUDOR domain-containing protein 7 (TDRD7) as a possible candidate acting in the initial stages of *R. philippinarum* germline differentiation. This is supported by literature data on LOTUS-TUDOR-containing proteins (comprising TDRD7 homologues), as well as by in situ localisation of TDRD7 in putative germ cells in their earliest stages of differentiation in *R. philippinarum* intestinal epithelium. Our interpretation is that TDRD7-immunolabeled cells within the intestinal epithelium would be Primordial Stem Cells (PriSCs), precursors of both Primordial Germ Cells (PGCs) and cells of the somatic lineage (Solana 2013). With in silico identification and literature data, we predicted that TDRD7 might be involved in early stages of germline differentiation, maybe in the formation of germ granules, and our histological observations in cellular populations previously determined as Vasph-tagged undifferentiated germ cells (Milani et al. 2015, 2017, 2018) provide good evidence for it. However, to solidly validate such hypotheses, future analyses including isoform characterization, immuno-TEM observations of germ granules, and functional tests in vitro are needed.

## Supplementary Information

Below is the link to the electronic supplementary material.

Supplementary file1 (PDF 4097 KB)

## Data Availability

The datasets generated during and/or analysed during the current study are available from the corresponding author on reasonable request.
