# Supplementary material for: Early germline differentiation in bivalves: TDRD7 as a candidate investigational unit for Ruditapes philippinarum germ granule assembly
Source: Histochem Cell Biol. 2021 Mar 26;156(1):19–34. doi: 10.1007/s00418-021-01983-0 (PMC8277629; doi:10.1007/s00418-021-01983-0)
Supplement: Supplementary file 1 — Supplementary file1 (PDF 4097 KB) [file 418_2021_1983_MOESM1_ESM.pdf]

Early germline differentiation in bivalves: TDRD7 as a candidate investigational unit for *Ruditapes philippinarum* germ granule assembly

Beatrice Filanti[1], Giovanni Piccinini[1], Simone Bettini[1], Maurizio Lazzari[1], Valeria Franceschini[1], Maria Gabriella Maurizii[1], Liliana Milani[1]*

[1]Department of Biological, Geological and Environmental Sciences - BiGeA, University of Bologna, Bologna, Italy

Beatrice Filanti and Giovanni Piccinini contributed equally

*Author for correspondence: Liliana Milani, E-mail: liliana.milani@unibo.it

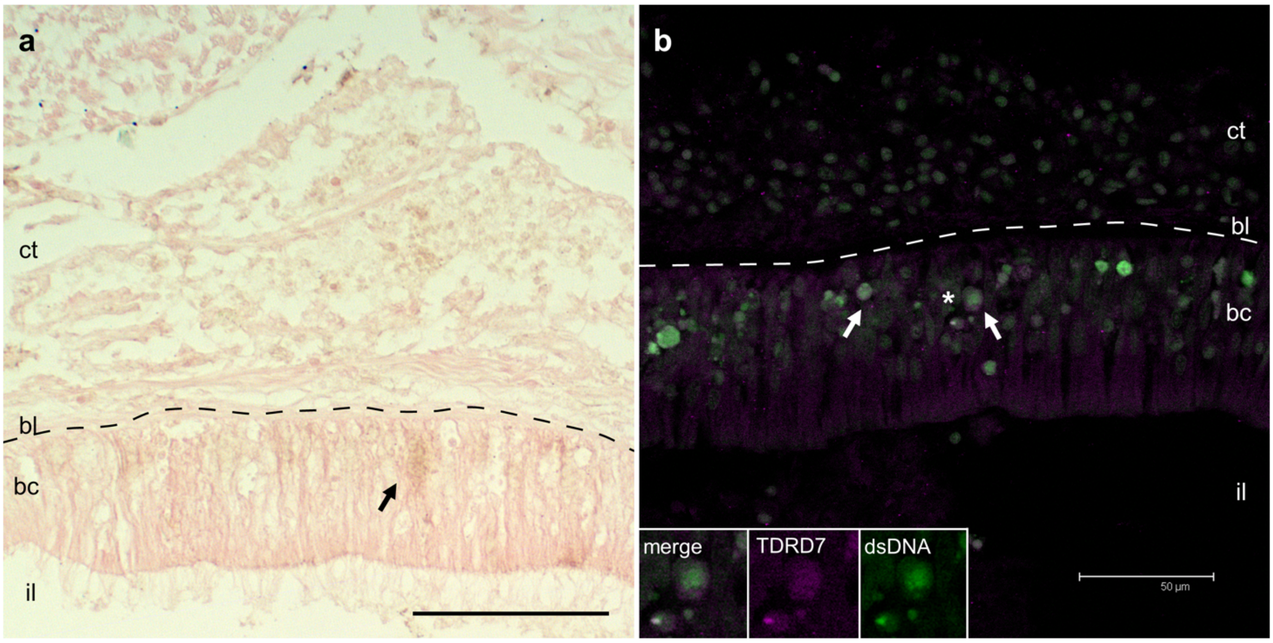

Supplementary Fig. 1 *Ruditapes philippinarum* tissues in spent phase stained with anti-TDRD7 by immunohistochemistry (IHC) and immunofluorescent (IF) assays. **a** IHC image where TDRD7-labeled cells are present in the intestinal epithelium and pointed out with the arrow. Brown: anti-TDRD7 staining. **b** Anti-TDRD7 staining was very light and restricted to few, small and round cells (arrows) between batiprismatic cells (bc) of the intestinal epithelium. No staining was observed in the connective tissue (ct). il = intestinal lumen; bl = basal lamina; asterisk = cells magnified in the inset. Magenta: anti-TDRD7 staining; Green: TO-PRO-3 nuclear dye. Scale bar: **a** = 100 µm; **b** = 50 µm.

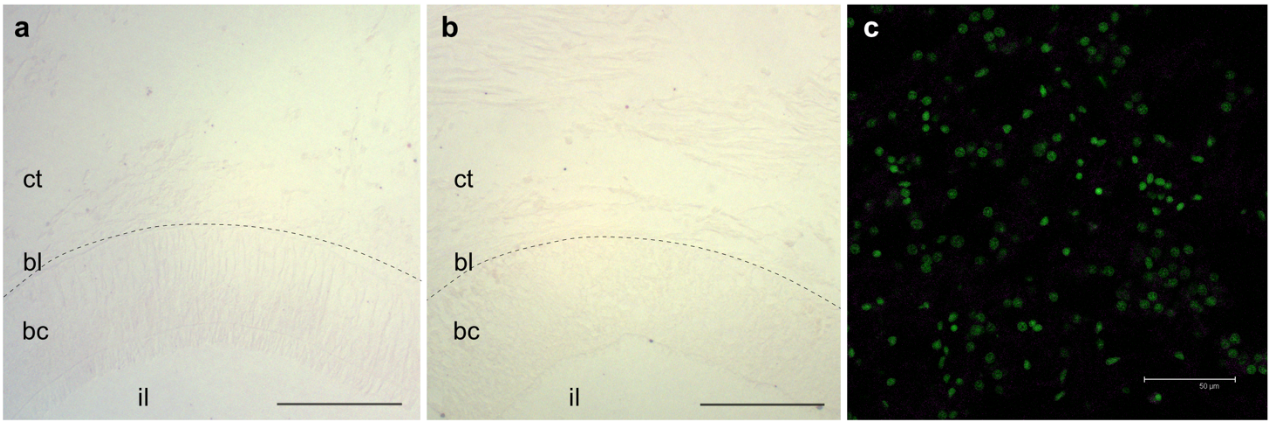

Supplementary Fig. 2 Control sections from individuals in seasonal sexual rest stained exclusively with secondary antibodies. **a** Section treated with HRP anti-rabbit (Santa Cruz Biotechnology Inc.). No staining was observed in the connective tissue (ct) and in cells between batiprismatic cells (bc). Brown: HRP anti-chicken staining (not visible); bl = basal lamina; il = intestinal lumen. **b** Section treated with the secondary antibody HRP anti-chicken (Santa Cruz Biotechnology Inc.). No staining was observed in the connective tissue (ct) and in cells between batiprismatic cells (bc) of the intestinal epithelium. Brown: HRP anti-chicken staining (not visible). **c** Portion of connective tissue treated with anti-chicken secondary antibody (DyLight 550). No staining was reported in somatic cells of the connective tissue (for other controls in the same species see also Ghiselli et al. 2019). Magenta: DyLight 550 anti-chicken secondary antibody (not visible); Green: TO-PRO-3 nuclear dye. Scale bar: **a**, **b** = 100 μm; **c** = 50 μm.

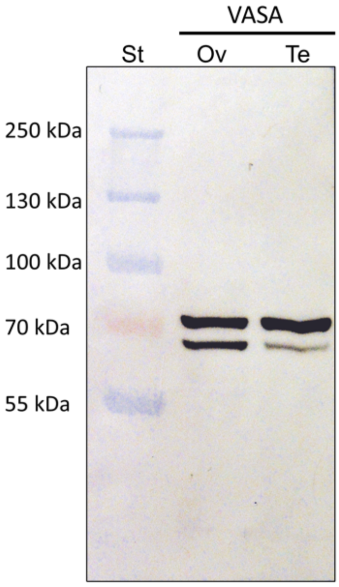

Supplementary Fig. 3 Western blot with anti-VASA/VAS (Abcam, ab209710, rabbit polyclonal antibody, diluted 1:2,000, loading 30 μg of total protein homogenate per lane). First lane F2, second lane M2 (specimens as in Supplementary Table 2). The resulted bands correspond with those previously obtained with antibodies specifically developed against *R. philippinarum* Vasa (anti-Vasph) (see Milani et al. 2015). The molecular weight of the protein standard (St; Thermo Scientific™ PageRuler™ Plus Prestained Protein Ladder) is reported in kDa on the left side of the panel.

Supplementary Table 1 PCR primers. All the 13 primer couples used are reported in the table. For the first and last sequence portion, we developed three couples since the *in silico* prediction had bad parameters and we wanted to be sure to amplify the portion: all six of them worked. The PCR cycle was: 94°C for 30s, 55°C for 30s; 73°C for 90s. This was repeated for a minimum of 30 and a maximum of 37 times, depending on the product yield. The reaction included 2 minutes at 94°C at the beginning and 5 minutes at 73°C at the end. The only difference between the reactions for the primer couples was the annealing temperature (see last column).

Name	Primer	Sequence	Length	Start	Stop	Annealing T (°C)
**1a**	Forward	GTCGTTCGAAAAGAGGCTGC	20	73	92	55
**1a**	Reverse	CTGTACTGACTTGAGCCCCG	20	631	612	55
**1b**	Forward	CGAAAAGAGGCTGCATTCGT	20	79	98	55
**1b**	Reverse	GGGGTAGAAATCGTGACCCG	20	609	590	55
**1c**	Forward	CGAAGTCGTTCGAAAAGAGGC	21	69	89	55
**1c**	Reverse	CCGTGGGGGTAGAAATCGTG	20	614	595	55
**2**	Forward	TCCACTTCACACATCCAGGC	20	507	526	55
**2**	Reverse	TCCCTACCGTCTCCTCCTTG	20	1242	1223	55
**3**	Forward	GCCATGTTGGAAGGTGGAGA	20	957	976	58
**3**	Reverse	CTCGGTGGACTGCTTGTTGA	20	1651	1632	58
**4**	Forward	AAGTACAATGAGGACCCGCC	20	1353	1372	58
**4**	Reverse	ATGGCCTGGCACGGATATTT	20	1849	1830	58
**5**	Forward	TCAACAAGCAGTCCACCGAG	20	1632	1651	55
**5**	Reverse	TGCTCTGGAATCAGCTCGTC	20	2401	2382	55
**6**	Forward	CCGTGATGCTCAGACTGGTT	20	2164	2183	55
**6**	Reverse	AATTTGCCGGACTCGGTGAT	20	2700	2681	55
**7**	Forward	GTTGCCGAGGTTGTTGACAG	20	2541	2560	55
**7**	Reverse	CCCGGACTTCGACCCAAAAT	20	3004	2985	55
**8**	Forward	ACACCGAGAACCGAATCACC	20	2667	2686	55
**8**	Reverse	CTGCCAAGTGTTGCGTTGAA	20	3418	3399	55
**9a**	Forward	GTCTGTTCCGAGACCATTCCA	21	3032	3052	55
**9a**	Reverse	TCTCGAAAGGAGTCTTTAGCAC	22	3701	3680	55
**9b**	Forward	CCGAGACCATTCCAGATTCCA	21	3039	3059	55
**9b**	Reverse	TTCTCGAAAGGAGTCTTTAGCAC	23	3702	3680	55
**9c**	Forward	CTGTTCCGAGACCATTCCAGA	21	3034	3054	55
**9c**	Reverse	CTCGAAAGGAGTCTTTAGCACTT	23	3700	3678	55

Supplementary Table 2 Summary of immunoblot analysis of anti-TDRD7 antibodies on testis and ovary extracts of adult *Ruditapes philippinarum* sampled at different stages of the gametogenic season. All male (M) and female (F) samples were blotted with both anti-TDRD7 antibodies (anti-EKF and anti-AYD).

	**Sample**	**Sampling date**	**Weight (kDa)**
**AYD**	M1	Early July	200+, 55
	M2	Early July	130, 60, 50, 37
	M3	Late July	50, 37
	M4	Late July	200, 180, 160, 130, 80
	M5	Late July	250
**EKF**	M1	Early July	~150
	M2	Early July	100, 70, 50, 37
	M3	Late July	37
	M4	Late July	200, 160, 120, 80
	M5	Late July	200
**AYD**	F1	Early July	200, 140, 100, 65, 55
	F2	Early July	60, 50, 37
	F3	Late July	37, 30
	F4	Late July	150, 70, 50, 37, 30
	F5	Late July	200, 150, 37
**EKF**	F1	Early July	150, 120
	F2	Early July	37
	F3	Late July	37, 30
	F4	Late July	150, 70, 50, 37, 30
	F5	Late July	37

REFERENCES

Ghiselli F, Maurizii MG, Reunov A, Ariño-Bassols H, Cifaldi C, Pecci A, Alexandrova Y, Bettini S, Passamonti M, Franceschini V, Milani L (2019) Natural heteroplasmy and mitochondrial inheritance in bivalve molluscs. Integrative and Comparative Biology 59:1016–1032. doi: 10.1093/icb/icz061

Milani L, Ghiselli F, Pecci A, Maurizii MG, Passamonti M (2015) The Expression of a Novel Mitochondrially-Encoded Gene in Gonadic Precursors May Drive Paternal Inheritance of Mitochondria. PLoS ONE 10(9):e0137468. http://doi.org/10.1371/journal.pone.0137468
